# Biochemical and molecular assessment of the superoxide dismutase (SOD) antioxidant enzyme regulation within *Medicago truncatula* root in response to iron deficiency

**DOI:** 10.1371/journal.pone.0335634

**Published:** 2025-10-31

**Authors:** Nadia Kallala, Wissal M'sehli, Manel Chaouachi, Karima Jalali, Ghassen Abid, Haythem Mhadhbi

**Affiliations:** Laboratory of Legumes and Sustainable Agrosystems (L2AD), Centre of Biotechnology of Borj Cedria (CBBC) BP. 901, Hammam-Lif 2050, Tunisia; Florida Agricultural and Mechanical University, UNITED STATES OF AMERICA

## Abstract

Iron (Fe) deficiency is a major nutritional stress affecting plant growth and metabolism. This study was conducted on three *Medicago truncatula* genotypes (TN8.20 and A17 as tolerant and T1.11 as a sensible genotype) cultivated in optimal and Fe-deficient conditions. Assessment of Fe deficiency effects was performed on some physiological and biochemical parameters with a particular focus on superoxide dismutase (SOD) activities and genes expression in roots. Our data showed that the sensitive genotype TN1.11 was more affected by Fe starvation compared to A17 and TN8.20. Overall, the relatively higher tolerance of A17 and TN8.20 to Fe deficiency was positively correlated to their ability to maintain higher plant biomass, Fe content, Fe use efficiency, Cu and Zn contents in roots. The oxidative stress associated with Fe-deficiency was evidenced by increased roots hydrogen peroxide (H_2_O_2_) levels, especially in TN1.11 genotype. In contrast, Assessment of SOD activity in roots revealed a significant increase in Cu/ZnSOD and MnSOD activities under Fe-deficient conditions, particularly in TN8.20. Gene expression analysis showed differential regulation of *FeSOD*, *Cu/ZnSOD* and *CHSOD* genes in response to Fe deficiency. Notably, TN8.20 exhibited upregulation of *Cu/ZnSOD* and down regulation of *CHSOD* under Fe-deficient conditions. TN8.20, which showed the highest SOD activities and gene expression levels, was identified as the most tolerant genotype. These findings highlight the physiological and molecular responses of *Medicago truncatula* to Fe deficiency and emphasize the comparative leaf-root analyses, revealing that SOD related genes in roots may serve as useful molecular markers for selecting Fe-deficiency-tolerant genotypes to cope with oxidative stress and nutrient imbalances.

## Introduction

Iron (Fe) is an essential micronutrient, indispensable for various physiological processes involved in plant growth and development, primarily as a cofactor for enzymes in vital metabolic pathways [1]. Despite its importance, Fe bioavailability in soil is often limited. The root system, as the primary organ responsible for nutrient uptake, adapts to iron deficiency through changes in roots morphology and physiology [2]. Under Fe deficiency, plants often exhibit a significant reduction in root biomass and primary root elongation [3]. To overcome Fe limitation, plants have developed sophisticated mechanisms to acquire and transport Fe [4]. Under Fe deficiency, they employ a series of morphological and physiological adaptations, especially in the root system, to enhance Fe transport and acquisition from the soil. For that, plants activate adaptive responses such as increased proton (H^+^) extrusion in rhizosphere to lower soil pH, stimulates Fe^3+^ reduction to the more soluble Fe^2+^ form and upregulation of Fe transporters to improve Fe solubilization and uptake [5]. These changes are particularly evident in strategy I plants, including dicots and non-graminaceous monocots, which rely on biochemical and structural modifications to improve Fe assimilation [6]. The uptake, utilization of Fe and the variations in pH of inter-root soil and in hormone concentrations in plants influence the interactions between various nutrients and their mineral homeostasis [7]. In terms of interactions between various elements, an antagonistic relationship exists among Fe, Zn and Cu, especially under iron deficiency, where these elements compete for uptake. Indeed, the uptake and accumulation of Mn, Cu and Zn are accelerated by iron deficiency [7,8].

Fe deficiency is frequently associated with oxidative stress in plants due to its critical role in electron transport, redox homeostasis and antioxidant enzyme activity. The excessive production of reactive oxygen species (ROS) under these conditions impairs several metabolic processes [9]. The uncontrolled accumulation of ROS can cause oxidative damage to essential biomolecules, including membrane lipids, thereby compromising cellular integrity and plant development [10].

To mitigate oxidative stress, plants rely on a complex antioxidant defense system that includes both enzymatic and non-enzymatic components. Among the enzymatic system, superoxide dismutase (SOD) plays a pivotal role as the first line of defense by catalyzing the dismutation of the highly reactive superoxide anion into H_2_O_2_ and O_2_ [11]. In plants, multiple SOD isoforms exist based on their metal cofactor composition and cellular localization including FeSOD, Cu/ZnSOD and MnSOD [12]. Notably, FeSOD activity is directly influenced by Fe availability, making Fe deficiency a dual stressor by simultaneously limiting antioxidant capacity while promoting ROS generation [13]. Fe serves as a cofactor for various antioxidant enzymes including superoxide dismutase (SOD), its deficiency can impair the function of this enzymatic antioxidant system leading to an increase in H_2_O_2_ [14]. Accordingly, gene expression levels of superoxide dismutase (SOD) isozymes (*MnSOD, Cu/ZnSOD,* and *FeSOD*) were studied to distinguish relationship between SOD expression and abiotic stress resistance [15]. Moreover, copper (Cu) chaperones play a crucial role in maintaining copper homeostasis in plant cells by binding and safely transporting copper ions to specific cellular compartments [16]. By regulating copper distribution, they prevent the generation of harmful radicals associated with free copper and ensure its proper utilization [17].

*Medicago truncatula,* a model legume species, is particularly affected by various biotic and abiotic stresses [18] including nutrient deficiencies [19,20]. Due to its small genome, its serves as an effective model for investigating the impact of Fe deficiency on plant behavior providing valuable insights into adaptive mechanisms. In previous studies [19], we found that the tolerance of two genotypes (A17 and TN8.20) was correlated with the maintenance of Fe content, the preservation of photosynthetic apparatus and an increased Cu content in leaves. These genotypes also showed an enhanced SOD activity and upregulation of Cu chaperone gene (*CHSOD*) expression in leaves. However, the effects of iron deficiency on SOD gene expression, Cu chaperone expression and their physiological consequences are still poorly understood due to the limited information on the specific molecular mechanisms of SOD isoform gene, particularly in roots, where is the first perception of Fe limitation. Since roots are directly exposed to Fe deficiency in the rhizosphere, explaining their specific responses is essential to clarify the physiological and molecular mechanisms underlying plant tolerance. According to Hua et al. [19], over 40% of the world’s soils suffer from severe Fe deficiency, with Mediterranean and Tunisian calcareous soils being particularly sensitive to this constraint [21], severely limiting plant growth and yield. Therefore, improving Fe use efficiency in plants is still original area of research, has become a global concern and a major focus of plant nutrition studies in recent years. In this context, we hypothesize that the Fe-deficiency induces specific regulatory mechanisms in *Medicago truncatula* roots involving changes in mineral nutrient homeostasis (Fe, Cu, and Zn), modulating the enzymatic activities of distinct SOD isoforms and transcriptional regulation of *FeSOD*, *Cu/ZnSOD*, and *CHSOD* genes. Our objective is to investigate root-specific responses to Fe-deficiency while establishing a direct comparison with leaf responses, thereby providing a complementary perspective and contributing to a more integrated understanding of whole-plant adaptive strategies to Fe stress.

## Materials and methods

### Plant material and Fe deficiency treatments

This study was conducted on three *Medicago truncatula* genotypes: Jemalong (A17), TN8.20 and TN1.11. Seeds were scarified and germinated according to Kallala et al. [19]. The germinated seeds were transferred in sterile Agir perlite moistened with distilled water for six days. After that, a similar sized seedlings was selected which are cultured as groups of 8 plants in 5 L of full strength modified nutrient solution containing macronutrients [22]. Three treatments were applied: Control (C: 50 µM Fe (III)-EDTA); Direct Fe-deficiency (DD: 5 µM Fe (III)-EDTA) and Induced Fe-deficiency by bicarbonate (ID: 50 µM Fe + 10 mM NaHCO_3_). Each treatment was performed with three independent biological replicates, and within each replicate, plants were grown in groups of eight. Four plants were harvested for plant biomass and four were used for enzymatic assays. The nutrient solution was refreshed every 7 days. Plants were cultivated in a growth chamber with a 16/8 h light/dark photoperiod at 18°C in the dark (RH of 60%) and 24°C in the light (RH of 80%).

### Plant biomass

After 21 days of treatment, leaves and roots were dried at 65°C for three days until a constant weight was achieved to determine their dry weight. Additionally, roots were frozen in liquid nitrogen and stored at 80°C for enzyme activity and molecular analysis. Relative growth rate (RGR) was calculated according to Kallala et al. [23].

### Relative root elongation

Relative root elongation (RRE) is a parameter used to asses root growth under experimental conditions compared to a control. RRE was calculated using the following formula: RRE= (Root elongation under stress condition/Root elongation under control condition)*100 [24].

### Extraction and determination of root Fe, Zn, and Cu contents

Subsamples (25 mg) of each dried root were digested with 20 mL of nitric acid/perchloric acid mixture (2.5:1, v/v) at 60°Cuntil complete dryness. The resulting extract was then dissolved in 20 mL of N/7HNO_3,_ distilled and filtered using Whatman paper. The final filtrates were analyzed for extractable Fe, Zn, and Cu using an atomic absorption spectrophotometer [25].

### Determination of Fe efficiency based on root dry weight, Fe use efficiency for dry weight, Fe uptake efficiency, Fe transport efficiency and Fe acquisition efficiency

Fe efficiency based on root dry weight (FeE), Fe use efficiency for dry weight (FeUE), Fe uptake efficiency (FeUp), Fe transport efficiency (FeT) and Fe acquisition efficiency (FeAE) was estimated using the following equations [26,27]:


$$\begin{array}{cc} & \text{Fe efficiency based on root dry weight FeE }(\%)\;=\;(\text{root dry weight under Fe}\text{-}\text{deficient conditions}/\;\\ & \text{root dry weight under Fe}\text{-}\text{sufficient conditions})*\text{100} \end{array}$$ (1)

$$\text{Fe use efficiency for dry weight FeUE }({\text{g mg}}^{-\text{1}})\;=\text{Dry weight}/\text{Fe}$$ (2)

$$\text{Fe uptake efficiency FeUp }({\text{mg g}}^{-\text{1}})\;=\;\text{Total Fe content}/\text{root dry weight}$$ (3)

Fe transport efficiency FeT (%)=(shoot Fe content/total Fe content)*100 (4)

$$\begin{array}{cc} & \text{Fe acquisition efficiency FeAE }(\%)=(\text{total Fe content under Fe}\text{-}\text{deficient conditions}/\\ & \text{total Fe content under Fe}\text{-}\text{sufficient conditions})*\text{100} \end{array}$$ (5)

### Hydrogen peroxide assay

Hydrogen peroxide content was determined using the KI method described by Velikova et al. [28]. Fresh root samples (500 mg, three replicates per treatment) were homogenised with 5 mL of 0.1% trichloroacetic acid (TCA). The homogenate was centrifuged at 12 000g for 15 min. Next, 0.5 mL of the resulting supernatant was mixed with 0.5 mL of 10 mM phosphate buffer (pH 7) and to1 mL of 1 M KI solution. The H_2_O_2_ concentration was measured spectrophotometrically at 390 nm, using a standard curve prepared with H_2_O_2_ solution as a reference.

### Superoxide dismutase (SOD) activities and protein determination

All operations were performed at 4°C to preserve enzyme activity. Root extracts were prepared by homogenising 200 mg of fresh roots in a mortar with 10% (V/V) polyvinylpolypyrrolidone (PVP) and 1 mL of 50 mM phosphate buffer (pH = 7.8) containing 0.1% (V/V) triton X-100 and 1 mM phenyl methylsulfonyl fluoride. The homogenate was then centrifuged at 13 000g for 20 min at 4°C and the supernatant was used for the determination of SOD activities according to the method described by Kallala et al. [19]. Protein content was determined using Bradford method [29] with bovine serum albumin as the standard.

### RNA extraction, cDNA synthesis, and real-time PCR assay

0.5 g of root samples were powdered in liquid nitrogen. Total RNA extracted from those samples that mixed with the extraction buffer as described by Chang et al. [30] with some modifications according to Abid et al. [31]. The extraction protocol was detailed by M’sehli et al. [20]. To estimate the RNA quality and quantity, a NanoDrop® ND1000 Spectrophotometer was used to measure the RNA concentration and an agarose gel (1.5%) was adopted. Then, the total RNA samples were treated with 5 U of RNase-free DNase I (Thermo Fisher Scientific) for 20 min at 37°C [32]. For real-time PCR analyses, 5 mg of total RNA was converted into cDNA using Turbo-I First Strand cDNA synthesis KIT (Biomatik) according to the manufacture’s protocol detailed by Kallala et al. [19].

### Statistical analysis

Statistical analysis were performed using SPSS program and means were separated according to the tukey test at p ≤ 0, 05. Data shown are means of three biological replicates of four technical replications for each treatment. Correlation matrix and heatmap were conducted using R software (Rx64 4.0.2). Hierarchical grouping *Medicago truncatula* genotypes based on their responses to Fe-deficiency was calculated based on Pearson correlations and complete linkage. Z-scores were calculated and used to generate the heatmap.

## Results

### Biomass and Relative growth rate

**Fig 1:**
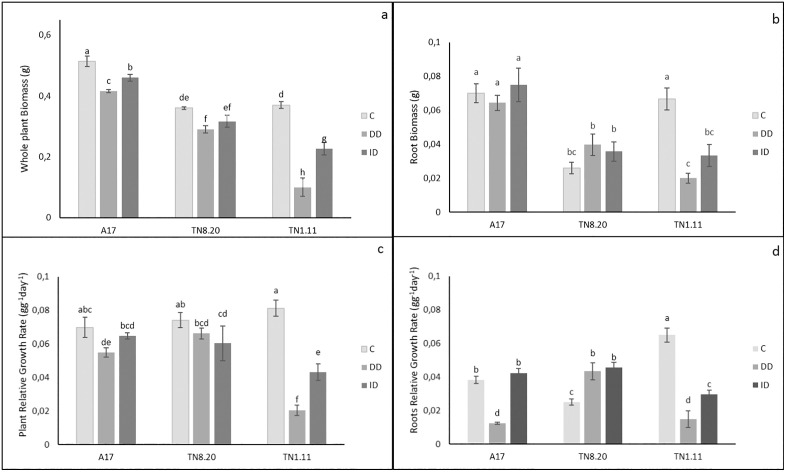
Effect of Fe availability on Plant biomass (a), root biomass (b), Relative growth rate of plant (c), Relative growth rate of roots (d) of three genotypes of *Medicago truncatula* grown in the presence of Fe (C), under Fe deficiency (DD) and induced Fe deficiency by bicarbonate (ID) during the treatment period (21 days). Bars are means ± SE. Values with the same letter per parameter are not significantly different according to Tukey test (p ≤ 0, 05), (n = 3).

Fig 1(a) illustrates the effect of iron deficiency on total plant biomass of *Medicago truncatula* genotypes. Results show a significant decrease of biomass in all genotypes under both direct (DD) and induced deficiency by bicarbonate (ID) conditions. A17 and TN8.20 showed the highest biomass production under iron deficiency compared to TN1.11 genotype. Specially, biomass decreased by 19% and 10% in A17, 19% and 13% in TN8.20 and 72% and 38% in TN1.11 under DD and ID conditions, respectively, compared to the control.

Fig 1(b) shows that in A17 and TN8.20 genotypes, root biomass remained relatively stable across all treatments, with no significant differences observed between control and iron deficiency conditions. In contrast, root biomass was significantly reduced under DD and ID conditions in TN1.11 genotype.

The relative growth rate (RGR) of whole plant decreased under both DD and ID conditions in three genotypes A17, TN8.20 and TN1.11, with the most pronounced reduction observed in TN1.11.Where RGR declined by 75% and 50%, compared to TN8.20 which showed a reduction of 10% and 18,9% under DD and ID conditions, respectively (Fig 1(c)).

Regarding RGR of root, results showed a decrease under DD in A17 (67%), and in TN1.11 under both DD (85%) and ID (55%) conditions. In contrast, A17 maintained a stable root RGR under ID, while the TN8.20 genotype exhibited a 72% increase in root RGR under DD and ID conditions compared to the control (Fig 1(d)).

### Length root and relative root elongation

**Fig 2:**
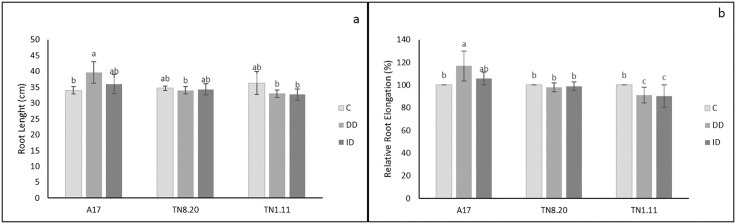
Effect of Fe availability on Root length (a) and relative root elongation (b) of three genotypes of *Medicago truncatula* grown in the presence of Fe (C), under Fe deficiency (DD) and induced Fe deficiency by bicarbonate (ID) during the treatment period (21 days). Bars are means ± SE. Values with the same letter per parameter are not significantly different according to Tukey test (p ≤ 0, 05), (n = 3).

Under direct iron deficiency (DD)**,** root length showed a significant increase in A17 genotype compared to the control, while no significant differences were observed in TN8.20 and TN1.11 genotypes under both treatments (Fig 2(a)). Similar results were observed for the relative root elongation parameter (RRE) (Fig 2(b)) with a significant increase in A17 under DD conditions.

### Fe content and efficiencies of utilization, uptake, transport and acquisition

#### Fe content.

Fe content decreased under iron deficiency treatments in *M. truncatula* genotypes. This decrease was more pronounced in the TN1.11 genotype, with reductions of 26% and 22% under DD and DI treatments, respectively, compared to the control (Table 1). In contrast, for the A17 and TN8.20 genotypes, the decrease in Fe content was less pronounced showing a reduction of 18% under iron deficiency conditions compared to the control.

**Table 1:** Effect of Fe availability on Cu, Zn, Fe contents, Fe use efficiency for dry weight (FeUE), Fe efficiency based on root dry weight (FeE), Fe uptake efficiency (FeUp), Fe transport efficiency (FeT) and Fe acquisition efficiency (FeAE) in roots of three genotypes of *Medicago truncatula* grown in the presence of Fe (C), under Fe deficiency (DD) and induced Fe deficiency by bicarbonate (ID) during the treatment period (21 days).

Genotype	Treatement	Fe (mg/gDW)	FeUE (g/mg)	FeE (%)	FeUp (mg/g)	FeT (%)	FeAE (%)	Cu (mg/g DW)	Zn (mg/g DW)
A17	C	3.06 ab	22.88 ab	100 bc	1.24 e	24.10 e	100 a	0.09 b	0.49 c
	DD	2.80 d	21.31 ab	78.0 c	1.24 e	24.17 e	91.7 b	0.15 a	0.63 b
	ID	2.98 bc	24.64 a	106 b	1.18 f	18.03 f	92.7 b	0.10 b	0.44 cd
TN8.20	C	3.17 a	8.38 c	100 bc	1.35 c	35.7 c	100 a	0.09 b	0.47 c
	DD	2.89 cd	13.81 bc	150 a	1.32 cd	32.3 cd	88.9 bc	0.15 a	0.58 b
	ID	2.60 e	14.07 bc	138.8 a	1.41 b	41.97 b	85.8 c	0.17 a	0.91 a
TN1.11	C	2.58 e	25.75 a	100 bc	1.52 a	51.83 a	100 a	0.08 b	0.16 e
	DD	1.92 g	10.33 c	32.7 d	1.45 b	45.31 b	71.13 d	0.15 a	0.66 b
	ID	2.11 f	15.71 bc	52.1 d	1.30 d	30.10 d	70.04 d	0.11 b	0.37 d

Values with the same letter per parameter are not significantly different according to Tukey test (p ≤ 0, 05), (n = 3).

#### Efficiencies of utilization, uptake, transport and acquisition.

In the A17 genotype, Fe efficiency based on root dry weight (FeE) and Fe use efficiency (FeUE) remains relatively stable under both DD and ID conditions (Table 1). On the other hand, the TN8.20 showed an increase of these parameters, while TN1.11 genotype showed a significant decrease under DD conditions.

Fe uptake efficiency (FeUp) decreases significantly under both DD and ID in TN1.11 and under ID conditions in A17 genotype condition. However, TN8.20 exhibits a marked increase in FeUp under ID conditions (Table 1).

In the A17 and TN8.20 genotypes, Fe Transport efficiently (FeT) remains relatively stable under DD conditions (Table 1). In contrast, under ID conditions, a significant decrease was observed in A17, while TN8.20 showed a significant increase. In the TN1.11 genotype, FeT significantly decreased under both DD and ID conditions.

Concerning Fe acquisition efficiency (FeAE), results showed a significant decrease under both DD and ID conditions in three genotypes. This decrease was more pronounced in TN1.11, with a reduction of 30% whereas in TN8.20 and A17, the decrease did not exceed 14.2% in under either conditions.

### Cu and Zn content

Regarding Cu content, the results show an increase in the roots of plants grown under Fe-deficient conditions (Table 1). This increase was more pronounced in the A17 and TN1.11 genotypes under DD conditions, and in the TN8.20 genotype under DI conditions, with respective increases of 59%. 93% and 94% compared to the control.

Similar results were observed for root Zn content in *M. truncatula* genotypes. A significant increase was detected in all three genotypes, with the increase being more pronounced in A17 and TN1.11 under DD conditions and in TN8.20 under DI treatment.

### H_2_O_2_ content

**Fig 3:**
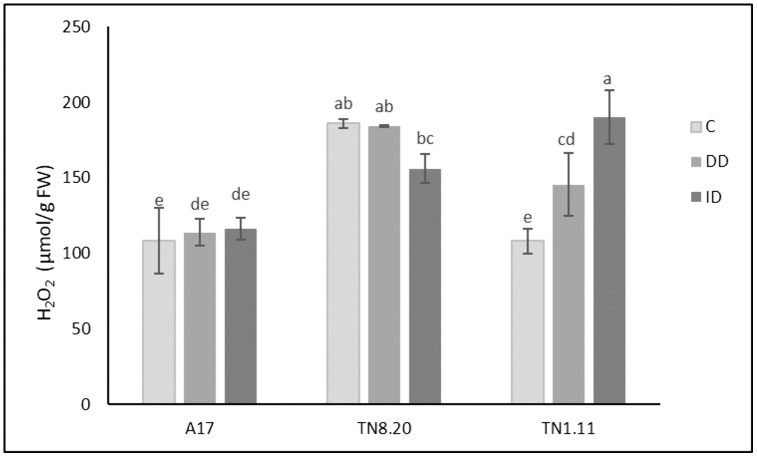
Hydrogen peroxide (H_2_O_2_) in roots of three genotypes of *Medicago truncatula* grown in the presence of Fe (C) under Fe deficiency (DD) and induced Fe deficiency by bicarbonate (ID) during the treatment period (21 days). Bars are means ± SE. Values with the same letter per parameter are not significantly different according to Tukey test (p ≤ 0, 05), (n = 3).

No significant increase in root H_2_O_2_ content was observed in A17 and TN8.20 under either DD or ID conditions (Fig 3). However, a significant increase in H_2_O_2_ content was recorded in TN1.11 under both DD and ID treatments, with increases of 34% and 76% respectively, compared to the control.

### SOD activity

The results revealed an increase in total SOD activity (SOD T) due to iron deficiency in the A17 and TN8.20 genotypes. In contrast, SOD activity decreased in TN1.11 under DD and ID treatments (Table 2).

**Table 2:** Enzymatic activity of total superoxide dismutase (SOD T) and its different isoforms (FeSOD), (Cu/ZnSOD) and (MnSOD) in roots of three genotypes of *Medicago truncatula* grown in the presence of Fe (C) under Fe deficiency (DD) and induced Fe deficiency by bicarbonate (ID) during the treatment period (21 days).

Genotype	Treatment	SOD T	Cu/ZnSOD	MnSOD	FeSOD
A17	C	65.08 d	10.50 c	43.59 f	10.99 bc
	DD	72.44 d	16.87 bc	52.38 ef	3.19 d
	ID	93.70 bc	14.31 bc	75.20 bc	4.19 d
TN8.20	C	97.57 bc	10.58 c	66.81 cd	20.19 a
	DD	105.79 b	18.71 b	80.19 b	6.88 cd
	ID	128.03 a	31.88 a	92.71 a	3.43 d
TN1.11	C	95.82 bc	11.53 c	70.41 bcd	13.89 ab
	DD	88.34 c	26.47 a	59.26 de	2.61 d
	ID	87.10 c	19.10 b	64.30 cd	3.70 a

Values with the same letter per parameter are not significantly different according to Tukey test (p ≤ 0, 05), (n = 3).

The results for SOD isoforms showed that Cu/ZnSOD and MnSOD activities were higher in the roots of genotypes A17. TN8.20 and TN1.11 grown under iron-deficient conditions compared to control conditions. However, these results did not apply to the MnSOD activity in TN1.11 under DD and ID conditions. Regarding FeSOD activity, the results indicated that this activity was significantly higher in control plants compared to deficient plants.

### SODs gene

**Fig 4:**
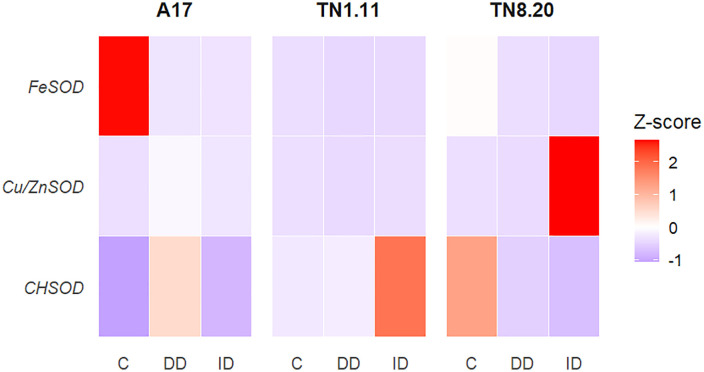
Heat map representation of the effects of Fe deficiency on the gene expression level in roots of three genotypes of *Medicago truncatula* grown in the presence of Fe (C) under Fe deficiency (DD) and induced Fe deficiency by bicarbonate (ID) during the treatment period (21 days), (n = 3).

The heatmap shows the expression levels (as Z-scores) of three superoxide dismutase (SOD) isoforms (*FeSOD*, *Cu/ZnSOD* and *CHSOD*) in the roots of *Medicago truncatula* genotypes (A17, TN8.20 and TN1.11) grown under Fe deficiency conditions (Fig 4). *FeSOD* gene expression was higher in A17 under control conditions. However, it was repressed in all genotypes of *M. truncatula* genotypes in response to iron deficiency conditions. In contrast. *Cu/ZnSOD* expression was significantly upregulated under induced Fe deficiency by bicarbonate (ID) in TN8.20 which showed the highest expression and under direct Fe deficiency (DD) in A17 genotype. As for the Cu chaperone gene (*CHSOD*), an induction of expression was observed in A17 and TN1.11. This induction was more important under TN1.11 under ID. However, this gene was repressed in the TN8.20 genotype under both DD and ID conditions.

### Correlation

**Fig 5:**
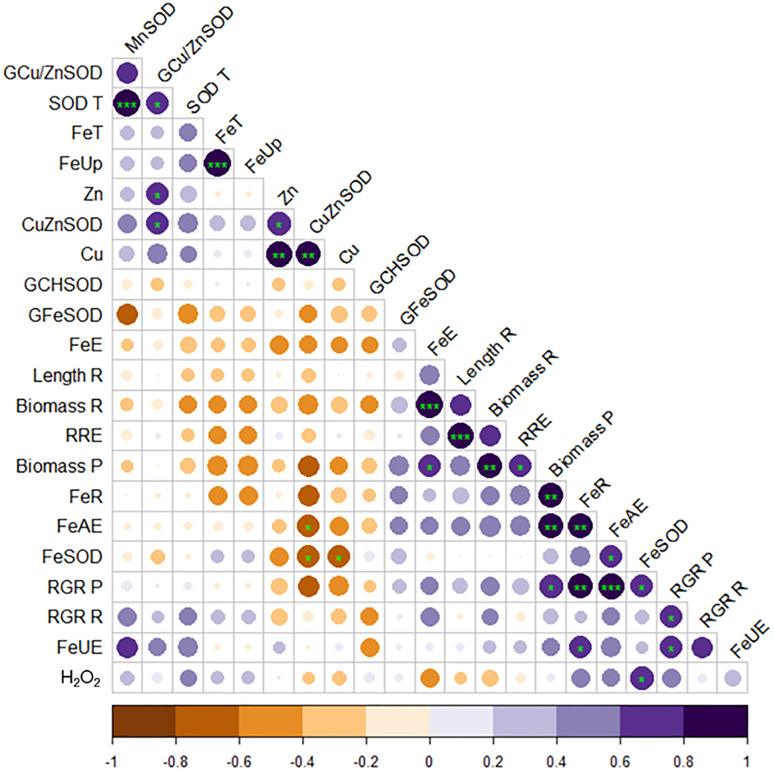
The Pearson correlation analysis of physiological, biochemical and molecular parameters of three genotypes of *Medicago truncatula* grown in the presence of Fe (C) under Fe deficiency (DD) and induced Fe deficiency by bicarbonate (ID) during the treatment period (21 days).

Pearson correlation analysis (Fig 5) highlights significant relationships among physiological, biochemical and molecular parameters in roots of *Medicago truncatula* genotypes under Fe-deficient conditions. Parameters with significant positive correlations are marked in dark violet, while those with negative correlations are marked in brown. The color intensity in the figure corresponds to the strength of the correlation. A significant positive correlation was observed between total SOD activity (SOD T) and its isoforms MnSOD and Cu/ZnSOD. Similarly, Fe uptake (FeUp) showed a significant positive correlation with Fe transport efficiency (FeT). Notably, root and plant biomass (Biomass R and Biomass P), relative root elongation (RRE), root Fe content (FeR), FeSOD activity and Fe acquisition efficiency (FeAE) were positively correlated. In contrast, Fe use efficiency (FeUE) displayed negative correlations with these growth and biochemical parameters. Additionally, Zn and Cu content in roots correlated positively with Cu/ZnSOD activity.

## Discussion

Our results demonstrated that iron deficiency negatively affected plant growth by reducing both biomass production and relative growth rate under conditions of direct (DD) and induced by bicarbonate (ID) iron deficiency across all genotypes. These findings are consistent with those of Truong et al. [33], who also reported that Fe deficiency reduces plant growth. Among the tested genotypes, A17 and TN8.20 maintained the highest biomass, indicating a degree of tolerance to iron deficiency. In contrast, TN1.11 exhibited the greatest reduction in biomass, suggesting that this genotype is particularly sensitive to iron deficiency. Regarding root biomass, A17 maintains root growth under iron stress, while TN8.20 displayed a significant increase in root biomass under both DD and ID conditions. In contrast, TN1.11 showed a significant reduction in root biomass under stress conditions compared to the control.

A similar pattern was observed for root length, which increased in A17, remained stable in TN8.20 but decreased in TN1.11. These results are positively correlated with relative root elongation (RRE). Consequently, the results highlight the greater tolerance of TN8.20 which adopts a strategy based on lateral root proliferation (increased biomass and relative root rate), whereas the tolerance of A17 is associated with increased primarily root length. This root growth response can be considered an adaptive mechanism. Indeed, iron deficiency leads to enhance main root growth, a reduction in root number, increased root hair density, higher root dry weight and greater root surface area [2]. Moreover, Divte et al. [34] demonstrated that the root system architecture plays a critical role in plant adaptation to abiotic stress and is essential for improving nutrient use efficiency, which aligns with the findings of the present study. In addition, previous studies showed that production of dry matter was in positive correlation with the ability to absorb more Fe from deficient soils [27,35].

In fact, the root Fe content is higher in TN8.20 and A17 under iron deficiency compared to TN1.11 genotype. In addition, the Fe efficiency based on root dry weight (FeE) and Fe use efficiency (FeUE) were higher in the TN8.20 genotype under iron deficiency, explaining the maintenance of plant growth. In contrast, these parameters decreased in TN1.11 which shows a significant decrease of FeUp, FeT and FeAE under both DD and ID conditions, indicating a reduced overall iron accumulation in the plant and it have a less efficient to maintain iron homeostasis or storage mechanism under stress. These findings suggest that each genotype has a distinct strategy for managing iron availability, with A17 and TN8.20 are capable of developing efficient mechanisms for Fe acquisition and TN1.11 exhibits a more sensitive response to iron deficiency. Moreover, Fe use efficiency (FeUE) showed a strong positive correlation with Fe concentration, confirming the strong correlation between iron availability, the plant’s capacity to efficiently utilize this micronutrient and plant biomass. This is explained by the fact that Fe is essential for plant growth and several vital processes [1]. TN8.20 which are better at maintaining overall iron content even under stress conditions and are able to adjust their active iron levels in response to Fe deficiency. It can be inferred that the greater accumulation of Fe in plants has induced the activation of the antioxidant system, as this element composes antioxidant enzymes [36].

Further analysis of Cu and Zn content showed a positive response under Fe deficiency. We noticed a higher increase of these metals concentration in roots of A17 and TN8.20 compared to TN1.11. Additionally, Cu content increased in the leaves of A17 and TN8.20, whereas it decreased in TN1.11 under Fe-deficient conditions (DD and ID). Zn content remained stable in TN8.20, while it shows a significant increase in A17 under DD and in TN1.11 under both DD and ID conditions [19]. These findings agree with an earlier study which demonstrated that iron deficiency in plants can significantly affect the uptake, transport and accumulation of micronutrients, particularly Zn and Cu [8]. In many species, such as soybean [8] and Arabidopsis [7], Fe deficiency leads to increase Zn uptake and accumulation, especially in roots. A significant part of the interactions between Fe and other micronutrients, such as Zn, Mn and Cu can be attributed to the competition among these ions for binding to transceptors or sensors [37]. This can be due to the upregulation of the metal transporters like IRT1 that induced under iron deficiency and can transport Zn. Also, some studies report increased Cu uptake and Cu shares transport pathways with Fe [38,39]. Rai et al. [39] showed that elevated Cu levels enhance the synthesis of Cu containing proteins which can functionally replace Fe dependent proteins.

The imbalanced of normal concentration range of these metals in plants causing various physiological implications. These implications trigger oxidative stress by promoting the generation of ROS [40]. In our study, we observed a significant increase of root H_2_O_2_ level in TN1.11 genotype under iron deficiency. Notably, H_2_O_2_ is increasingly recognized as a key signaling molecule involved in plant stress responses [41]. One of the primary responses to Fe deficiency is the enhanced production of ROS, which induced oxidative stress. The excessive accumulation of ROS disrupts cellular homeostasis, compromising membrane integrity, proteins and various biomolecules [42].

To cope with elevated ROS levels, plants depend on an efficient antioxidant system to mitigate stress effects and repair oxidative damage. Our previous studies showed that these genotypes active antioxidant enzyme such as catalase (CAT) and gaϊacol peroxidase (POX) in both of leaves and roots [23], as well as SOD activity specially in leaves under iron deficient conditions [19]. Our study are confirmed by other research that showed that iron deficiency induced SOD activity in tolerant plants [43]. Similarly, transgenic *Arabidopsis* plants overexpressing MxbHLH30 exhibited increased SOD activity under Fe stress [7]. Our results showed that total SOD activity in roots was higher in the TN8.20 and A17 genotypes. This increase was positively correlated with Cu/ZnSOD and MnSOD activities. Similar patterns were observed in the leaves of these two tolerant genotypes, indicating an important induction of Cu/ZnSOD and MnSOD under Fe-deficiency [19]. Furthermore, recent studies confirm that Fe limiting conditions decreased FeSOD activity but a compensatory increase of Cu/ZnSOD and MnSOD isoforms activities in plants [44]. Similarly, soybean plants with contrasting iron deficiency exhibit differential regulation of SOD isoenzyme activities, with Fe-efficient plants maintaining superior management of oxidative stress [13]. The increase in SOD activity following Fe supplementation could be primarily due to the enhanced abundance of Cu/ZnSOD and MnSOD [44].

Iron deficiency in plants, which subsequently activates antioxidant systems to mitigate oxidative stress, also strongly influences the expression of various SOD isoform gene. In roots of the three *Medicago truncatula* genotypes, *FeSOD* gene expression was significantly repressed. In contrast, *FeSOD* expression was induced in leaves of A17 and TN1.11 genotypes under induced Fe deficiency by bicarbonate (ID) and direct Fe deficiency (DD), respectively [19]. These results indicating that root tissues are more responsive to Fe deficiency in terms of *FeSOD* gene activation. This suggests a localized antioxidant response in roots to counteract the oxidative stress induced by iron deficiency [45]. The expression of SOD isoforms is closely regulated by the availability of their respective metal cofactors. Indeed, results showed a high induction of *Cu/ZnSOD* gene expression in roots of TN8.20 under induced Fe deficiency by bicarbonate (ID), whereas a contrasting response was observed in the leaves [19]. Moreover, the presence of Fe up-regulated the expression levels of *FeSOD*, *Cu/ZnSOD*, *MnSOD* gene in grass species. These findings suggest that Fe regulates SOD activity in both chloroplasts and mitochondria at transcriptional and protein levels, contributing to the scavenging of O_2_^•−^ in stressed leaves [44]. Also, in *Arabidopsis thaliana*. Fe deficiency leads to the up-regulation of *Cu/ZnSOD* genes (CSD1 and CSD2), while a down-regulating *FeSOD* genes (FSD1 and FSD2) was observed [46]. This contrasting gene expression pattern represents a coordinated adaptive response to Fe limitation, involving enhanced copper accumulation in the rosettes to maintain antioxidant defense systems. Furthermore, the expression of *FeSOD* genes is influenced by the availability of other metals, such as copper. This regulatory mechanism enables plants to adapt to fluctuations in metal availability by modulating the expression of specific SOD isoforms. For that, the Cu chaperone gene (*CHSOD*) was analyzed. Results showed that *CHSOD* expression was induced in A17 under DD and in TN1.11 under ID, while it was repressed in TN8.20 genotype under both DD and ID conditions. In contrast, *CHSOD* expression was induced in leaves of TN8.20 [19]. These contrasting expression patterns observed between roots and leaves on TN8.20 suggest a tissue specific regulation of oxidative stress responses. In roots, the induction of *Cu/ZnSOD* coupled with the repression of *CHSOD* gene indicates a prioritization of antioxidant defense mechanisms over Cu transport and storage. In contrast, the coordinated induction of *CHSOD* expression, enhanced Cu/ZnSOD activity and increased Cu content in leaves emphasizes the importance of maintaining both Cu homeostasis and ROS-scavenging capacity to protect photosynthetic metabolism under Fe-deficiency [47]. Indeed, these chaperones interact with copper transporters and other proteins to coordinate copper allocation. particularly under abiotic stress conditions leads to oxidative stress [48]. Moreover, copper chaperones contribute to the regulation of antioxidant enzymes, thereby supporting the plant’s defense against oxidative damage [49]. In *Arabidopsis thaliana*, the copper chaperone plays a key role in coordinating both iron and copper responses. Under Fe deficiency, plants reduced copper chaperone gene to exhibited an altered growth responses [50]. Overexpression of high-affinity copper transporters in *Arabidopsis thaliana* disrupts iron homeostasis, resulting in altered expression of iron uptake genes and their regulatory transcriptional factors. This can also result from the accumulation of iron (III) which suppresses local iron deficiency responses and ultimately leads to a reduced overall iron content in the seedlings [51].

## Conclusion

**Fig 6:**
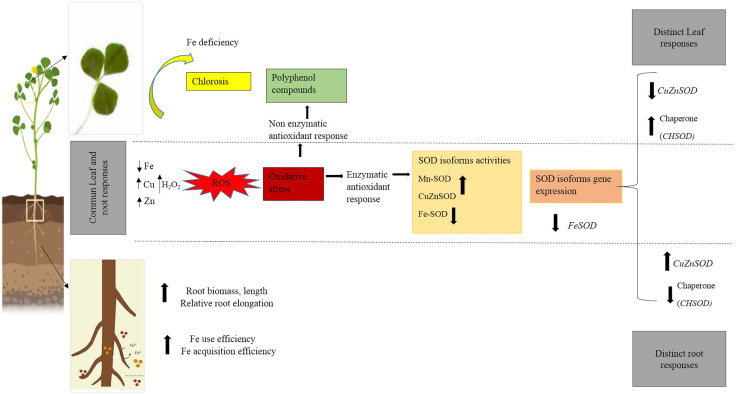
Integrative comparative study of leaf and root adaptive responses to Fe deficiency in tolerant *Medicago truncatula* genotype.

This study provides a comprehensive overview of the physiological and molecular mechanisms underlying Fe deficiency tolerance in *Medicago truncatula roots.* Taken together with our previous finding in leaves parts, the results revealed that genotypes adopt distinct strategies to cope with Fe deficiency, displaying tissue-specific responses in leaves and roots. The tolerant genotype TN8.20 maintains root growth under Fe-deficiency conditions by enhancing Fe acquisition and utilization efficiency, while also activating root antioxidant defenses through increased activity of SOD isoforms (Fig 6). Notably, both leaves and roots exhibit similar patterns in mineral accumulation and SOD activities, indicating a coordinated systemic response. However, contrasting expression patterns of *Cu/ZnSOD* under both Fe deficiency treatments (DD and ID) and of *CHSOD* genes under the ID treatment were observed between leaves and roots. Overall, tolerance to Fe deficiency relies on a coordinated network of root morphological adaptation, metal homeostasis and antioxidant regulation across tissues, providing key for breeding Fe efficient legumes.

## Supporting information

S1 File Analysis data. (XLSX)
